# Recent advances in gut microbiota-mediated regulation of fat deposition and metabolic disorders

**DOI:** 10.20517/mrr.2025.25

**Published:** 2025-08-20

**Authors:** Xiaoyan Cui, Qianwen Yuan, Jiali Long, Jiaxin Zhou

**Affiliations:** College of Animal Science and Technology, Yangzhou University, Yangzhou 225009, Jiangsu, China.

**Keywords:** Gut microbiota, fat deposition, short-chain fatty acids, bile acids, metabolic disorders, chicken, obesity

## Abstract

The gut microbiota critically regulates lipid metabolism through microbial metabolites and host signaling pathways. Short-chain fatty acids (SCFAs), derived from dietary fiber fermentation, suppress hepatic lipogenesis via inhibition of SREBP-1c and enhance mitochondrial β-oxidation through GPR41/43 activation. Microbial enzymes convert primary bile acids into secondary bile acids, which activate FXR to inhibit lipogenesis and TGR5 to promote adipose thermogenesis. Lipopolysaccharide (LPS) from dysbiotic microbiota triggers TLR4-NF-κB signaling, exacerbating insulin resistance and adipose inflammation. Branched-chain amino acids (BCAAs), metabolized by gut microbes, drive adipogenesis via mTORC1-PPARγ signaling, with elevated circulating BCAAs linked to obesity. In livestock, microbiota modulation optimizes fat deposition: probiotics in pigs enhance intramuscular fat via *Lactobacillus*-enriched communities, while dietary succinate or coated sodium propionate reduces abdominal fat in broilers by reshaping cecal microbiota. Fecal microbiota transplantation confirms microbial causality in transferring fat phenotypes. Dysbiosis-associated mechanisms are conserved across species, where SCFAs and bile acids ameliorate metabolic inflammation, whereas LPS and BCAA imbalances worsen lipid dysregulation. Metabolic disorders, including obesity, type 2 diabetes (T2D), and non-alcoholic fatty liver disease (NAFLD), are tightly linked to gut microbiota perturbations. Dysbiosis drives LPS translocation and barrier impairment. These changes, along with altered metabolites, promote inflammation and fat deposition. Future strategies should integrate multi-omics and precision engineering of microbial consortia to advance therapies for both livestock and human metabolic health.

## INTRODUCTION

The gut microbiota, a dynamic ecosystem of trillions of bacteria, archaea, viruses, and fungi, orchestrates host metabolism and energy homeostasis through its expansive metabolic repertoire^[1,2]^. Dysbiosis, characterized by shifts in community composition and function, has been strongly linked to aberrant fat deposition and metabolic disorders such as obesity, type 2 diabetes (T2D), and non-alcoholic fatty liver disease (NAFLD)^[3]^. Emerging evidence highlights the gut-liver axis, microbial metabolites including short-chain fatty acids (SCFAs), bile acids, and lipopolysaccharide (LPS), and immune crosstalk as central mediators of lipid metabolism and inflammation^[4,5]^. Germ-free and antibiotic-treated murine models have been foundational: colonizing adult germ-free C57BL/6 mice with conventional microbiota induces up to a 60% increase in body fat and insulin resistance within two weeks despite reduced caloric intake, establishing a causal link between microbes and fat deposition^[1]^. Mechanistic studies reveal that gut microbes enhance monosaccharide absorption and *de novo* hepatic lipogenesis via suppression of fasting-induced adipose factor and increased adipocyte lipoprotein lipase (LPL) activity^[6]^. Moreover, fecal microbiota transplantation (FMT) from obese donors transfers adiposity to germ-free or antibiotic-treated recipients, while lean donor FMT protects against high-fat-diet-induced weight gain^[1,7]^. An elevated Firmicutes/Bacteroidetes ratio commonly observed in obese states correlates with enhanced caloric extraction from complex polysaccharides and increased fat mass in both mice and humans^[3]^.

Concurrently, livestock research has harnessed microbiota manipulation to optimize fat deposition for meat quality without compromising growth performance^[8]^. In pigs, dietary interventions such as L-glutamate supplementation in Shaziling breeds and targeted probiotic administration have increased intramuscular fat (IMF) and modulated backfat thickness through specific shifts in microbial taxa^[9]^. In broilers, succinate and coated sodium propionate reshape cecal communities to reduce abdominal fat and enhance carcass traits^[10,11]^. Furthermore, dietary components (e.g., fibers, probiotics) directly shape microbial composition and function, establishing diet as a primary modulator of microbiota-mediated lipid metabolism across species^[12,13]^. Together, these integrative findings from murine and agricultural models underscore the translational potential of microbiota-based interventions for controlling fat deposition and mitigating metabolic disorders across species^[14]^.

## MICROBIAL COMPOSITION AND FAT DEPOSITION

### Obesity-associated dysbiosis in murine models

Obese murine models, including genetically obese ob/ob mice and those fed a high-fat diet (HFD), consistently exhibit an elevated Firmicutes/Bacteroidetes ratio compared to their lean counterparts, suggesting that this microbial shift enhances dietary energy harvest and promotes lipogenesis^[15,16]^. In particular, the expansion of Firmicutes observed in ob/ob mice is associated with increased production of SCFAs, such as acetate, propionate, and butyrate, which activate G protein-coupled receptors (GPCRs) GPR41 and GPR43 on adipocytes^[17]^. This activation contributes to adipocyte hypertrophy and greater fat storage by influencing host lipid metabolism and hormonal regulation^[13,18]^. On the other hand, dietary supplementation with fermentable fibers such as resistant starch and inulin has been shown to remodel the gut microbiota toward SCFA-producing Bacteroidetes, resulting in decreased fat mass and improved glucose tolerance in obese mice^[19,20]^, thus indicating a reversible aspect of diet-induced dysbiosis. Further supporting the causal role of the microbiota in fat deposition, studies using germ-free C57BL/6 mice, which lack all microbial colonization, have shown that these mice accumulate approximately 40% less total body fat compared to conventional mice, despite consuming more calories^[1]^. This finding implies that the presence of gut microbiota facilitates more efficient extraction of dietary energy and promotes fat accumulation^[18]^.

Similarly, antibiotic treatment of conventional mice leads to reduced weight gain under HFD conditions, and the recolonization of germ-free or antibiotic-treated mice with microbiota derived from either obese or lean donors successfully transfers the corresponding adiposity phenotypes to the recipients, thereby establishing a direct causal link between gut microbial composition and host fat accumulation^[18,21]^. FMT experiments provide additional evidence for this causality: germ-free or antibiotic-treated mice that receive fecal material from obese donors develop increased white adipose tissue mass, enlarged adipocytes, and impaired insulin sensitivity^[1,7]^. In contrast, transplantation with microbiota from lean donors protects mice from HFD-induced obesity and its associated metabolic disturbances^[18,21]^. These observations highlight the critical influence of microbiota-derived signals on systemic energy metabolism. Beyond the structural composition of the gut microbiota, specific microbial metabolites serve as key effectors that modulate host lipid metabolism via diverse signaling pathways. Acetate, one of the primary SCFAs, acts as a substrate for hepatic *de novo* lipogenesis, whereas propionate has been found to inhibit hepatic cholesterol synthesis. Butyrate, in turn, plays a distinct role by enhancing mitochondrial function in brown adipose tissue, thereby promoting fatty acid oxidation and thermogenesis, which contributes to increased energy expenditure^[13,22]^. Collectively, the findings from these murine studies illustrate a complex and robust mechanistic framework in which dysbiosis, characterized by an elevated Firmicutes/Bacteroidetes ratio, increased SCFA production, and shifts in microbial metabolic output, directly contributes to pathological fat accumulation and metabolic dysfunction. This body of evidence underscores the gut microbiota’s essential role in regulating fat deposition and its potential as a therapeutic target for obesity and related metabolic disorders.

### Specific microbiota in pigs and chickens

Distinct pig breeds harbor characteristic gut microbiota compositions that are closely associated with breed-specific fat deposition traits^[23,24]^. For instance, the Chinese indigenous Ningxiang pig, known for its high IMF content, possesses a cecal microbiota enriched in *Lactobacillus* and branched-chain amino acid (BCAA) metabolic pathways^[25]^. Transplantation of *Lactobacillus reuteri* isolated from Ningxiang pigs into lean Duroc × Landrace × Yorkshire (DLY) pigs or rats significantly increases circulating BCAA levels and IMF accumulation, highlighting a causal role for this microbial consortium in lipid deposition^[23,25]^. Moreover, FMT from obese Ningxiang pigs into lean DLY pigs remodels the recipient’s gut microbial community and downregulates the expression of the carnitine transporter SLC22A5 in skeletal muscle, thereby reducing fatty acid oxidation and promoting lipid accumulation^[23]^. Comparative analyses further reveal that native breeds such as Laiwu or Tibetan pigs display higher microbial diversity and unique taxa enriched in lipid-associated pathways compared to commercial breeds (e.g., Duroc, Landrace, Large White), which tend to harbor gut communities dominated by carbohydrate-fermenting bacteria (e.g., *Clostridium*, *Catenibacterium*) and methanogens - features that may underlie differences in energy utilization efficiency and backfat thickness^[26,27]^. Additionally, gut microbiota composition undergoes dynamic shifts across developmental stages, with lactating piglets dominated by lactic acid bacteria and *Bifidobacterium*, while post-weaning stages show a rapid increase in fiber-degrading genera such as *Prevotella* and *Roseburia*, which in turn modulate host lipid metabolism^[28,29]^. Importantly, gut microbiota appears to regulate fat deposition in a depot-specific manner; certain microbial taxa and metabolites (e.g., SCFAs) influence subcutaneous fat (e.g., backfat thickness) and IMF differently, through modulation of host lipid metabolism genes such as LPL and ANGPTL4^[29]^. For example, obese-type pigs exhibit elevated LPL expression and reduced ANGPTL4 levels in muscle, promoting IMF accumulation, while *Lactobacillus reuteri* from Ningxiang pigs modulates SLC22A5-mediated carnitine transport to enhance IMF specifically^[23,29]^. Together, these findings underscore the breed-, stage-, and depot-specific roles of the gut microbiota in regulating porcine fat deposition and support the development of precision microbiota-targeted strategies in swine production.

Emerging evidence from poultry research underscores the pivotal role of gut microbiota in modulating fat deposition, largely independent of host genetic background [Table 1]. For instance, specific microbial taxa, including *Methanobrevibacter* and *Mucispirillum schaedleri*, exhibit significant correlations with adipose tissue accumulation in chickens^[30]^. Further investigations reveal that cecal microbiota may modulate abdominal fat deposition through lipid metabolism pathways. Notably, the relative abundance of *Parabacteroides*, *Parasutterella*, *Oscillibacter*, and *Anaerofustis* shows a positive association with fat deposition, whereas *Sphaerochaeta* demonstrates an inverse relationship^[31]^. Additionally, age-dependent dynamics in abdominal fat development correlate with shifts in gut microbiota composition. Studies indicate that *Coprobacillus*, *Shigella*, and *Butyricicoccus* are negatively associated with propionic acid, butyric acid, and abdominal fat mass but positively correlate with isobutyric acid levels^[32]^. In broilers, dietary succinate (0.4%) reduced abdominal fat deposition by enriching beneficial cecal microbes (e.g., *Blautia*, *Sellimonas*) and altering amino acid metabolism linked to lipid handling^[10]^. Coated sodium propionate supplementation similarly inhibited fat deposition and reduced feed intake, accompanied by decreased adipocyte size and modulation of gut microflora, highlighting the role of propionate as a microbiota-mediated feed additive^[11]^. Dietary folic acid at 13 mg/kg decreased abdominal fat and increased SCFA-producing taxa, suggesting that vitamin-microbiota synergy can fine-tune carcass composition in broilers^[33]^. Dietary fiber treatment reduced abdominal fat and altered gut microbiota in yellow-feathered broilers fed corncob meal, decreasing *Phascolarctobacterium*, Rikenellaceae, and *Faecalibacterium* while increasing *Akkermansia*^[12]^. Studies utilizing FMT demonstrated that folic acid supplementation mitigates abdominal adipose accumulation in broilers, a process potentially mediated by gut microbial shifts. LEfSe analysis identified *Lactobacillus*, *Clostridium*, and *Dehalobacterium* as dominant taxa in the folic acid-treated group, suggesting their role in this regulatory mechanism^[34]^. Furthermore, dietary inclusion of fermented grape seed meal enhances broiler growth performance while suppressing abdominal fat deposition, likely via modulation of intestinal microbial communities^[35]^. In parallel, phytosterol supplementation alters gut microbiota composition in broilers, characterized by reduced bacterial alpha diversity and a marked increase in probiotic populations such as *Lactobacillus* within intestinal digesta^[36]^. In addition, correlation analysis revealed that many Firmicutes members had a highly positive relationship with blood lipid levels and fat storage capacity, which might contribute to the lower abdominal fat phenotype^[37-39]^. These findings collectively demonstrate that targeted modulation of gut microbiota through diet or microbial interventions offers a promising strategy for controlling fat accumulation in broilers.

**Table 1:** Microbial taxa reported to affect abdominal fat deposition in chickens in the past five years

**Influence factors**	**Sampling ages and breeds**	**Phenotype**	**Related gut microbial taxa**	**Ref.**
Succinate	21-day-old yellow-feathered broiler	Abdominal fat deposition	*Blautia* and *Sellimonas*	Wang *et al.* (2024)^[10]^
Coated sodium propionate	42-day-old broiler		*Alistipes*, *Lactobacillus*, *Bifidobacterium*, Lachnospiraceae and *Helicobacter*	Wang *et al.* (2021)^[11]^
Corncob meal	135-day-old yellow-feathered broiler		*Akkermansia*, *Phascolarctobacterium*, Rikenellaceae, *Faecalibacterium*	Cui *et al.* (2022)^[12]^
Host genetics	78-day-old yellow-feathered broiler		*Methanobrevibacter*, *Mucispirillum schaedleri*	Wen *et al.* (2019)^[30]^
Higher and lower abdominal fat	1, 4, and 12 months of age turpan cockfighting × white leghorn		*Sphaerochaeta*, *Parabacteroides*, *Parasutterella*, *Oscillibacter*, *Anaerofustis*	Chen *et al.* (2023)^[31]^
Age-associated changes	14, 28, and 42-day-old broiler		*Coprobacillus*, *Shigella*, *Butyricicoccus*	Liu *et al.* (2023)^[32]^
Folic acid	28-day-old broiler		*Alistipes*, *Oscillospira*, *Ruminococcus*, *Clostridium*, *Dehalobacterium*, *Parabacteroides*	Liu *et al.* (2023)^[33]^
Folic acid	28-day-old broiler		*Lactobacillus*, *Clostridium*, *Dehalobacterium*	Liu *et al.* (2024)^[34]^
Fermented grape seed meal	56-day-old yellow-feathered broiler		Bacteroidetes, Firmicutes	Nan *et al.* (2022)^[35]^
Phytosterols	42-day-old broiler		*Lactobacillus*	Dai *et al.* (2023)^[36]^
Corn resistant starch	21-day-old broiler		Bacteroidetes, Firmicutes	Zhang *et al.* (2020)^[37]^
Anoectochilus roxburghii extract	63-day-old yellow-feathered broiler		Bacteroidetes, Firmicutes	Wu *et al.* (2023)^[38]^
Echinocystic acid	1-day-old K901 broiler		Bacteroidetes, Firmicutes	Xiao *et al.* (2025)^[39]^
Lactococcus G423	21, and 42-day-old broiler		*Lactobacillus*, Firmicutes	Wang *et al.* (2024)^[40]^
Lean- and fat-line broilers	49-day-old broiler		*Escherichia coli*, *Candidatus Acetothermia bacterium*, *Alistipes sp*, *Ruminococcaceae bacterium*, *Clostridiales bacterium*, *Anaeromassilibacillus sp.*	Jing *et al.* (2021)^[41]^
Obese and lean chickens	160-day-old broiler (shouguang, luqin)		*Erysipelatoclostridium*	Liu *et al.* (2022)^[42]^
Two native breeds	42-day-old AA and 82-day-old beijing-you broiler		*Lactobacillus*	Lei *et al.* (2022)^[43]^
Different abdominal fat deposition	125-day-old tiannong partridge chicken		Bacteroidetes, Firmicutes, *Parabacteroides*, *B. salanitronis*, *B. fragilis*, *P. distasonis*, *Olsenella*, *Slackia*, *Methanobrevibacter*	Xiang *et al.* (2021)^[44]^

## MECHANISMS OF MICROBIOTA-MEDIATED LIPID METABOLISM

### SCFAs

**Figure 1:**
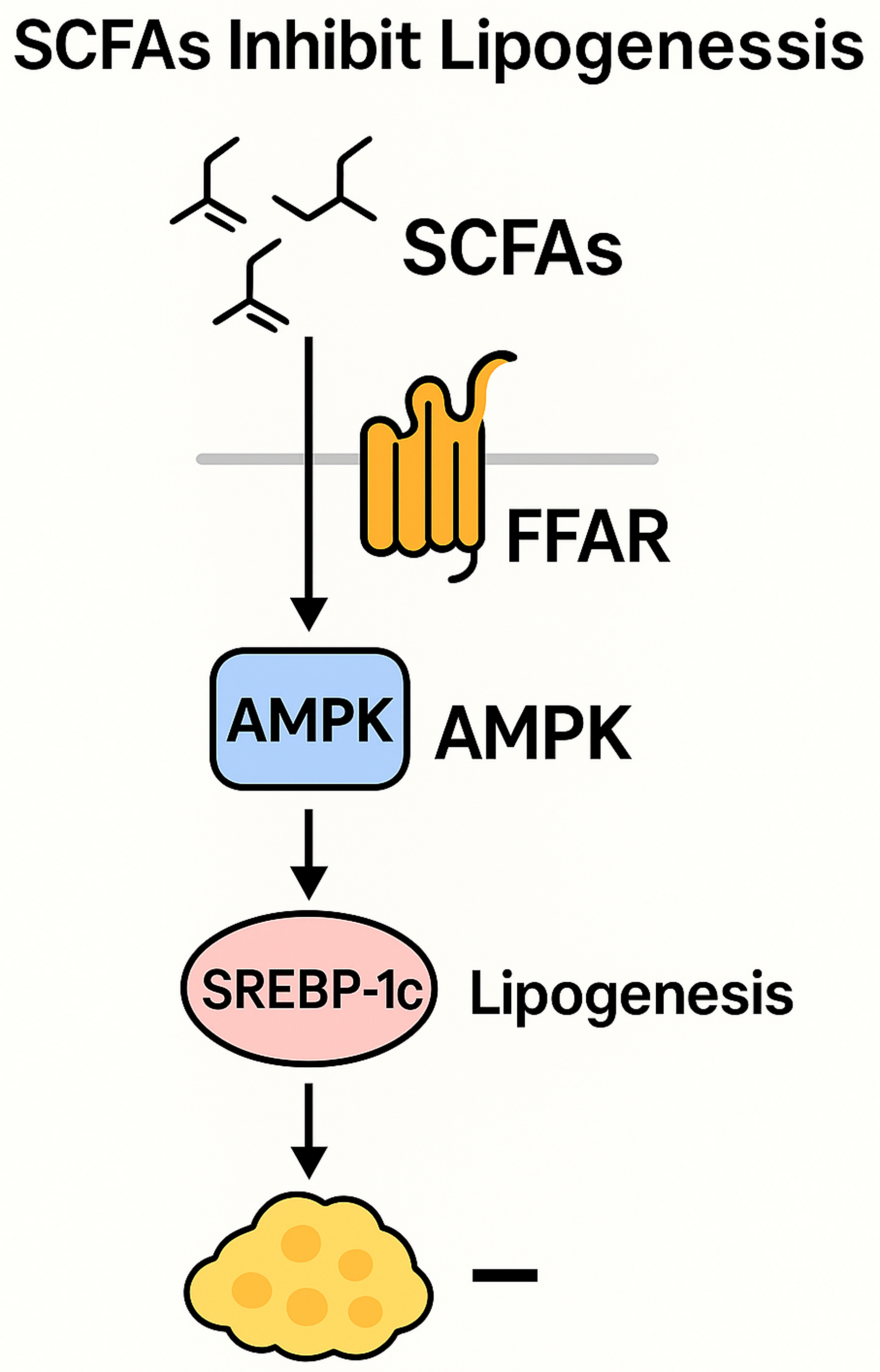
Signaling pathway diagram of SCFAs-mediated inhibition of adipogenesis. SCFAs: Short-chain fatty acids.

**Figure 2:**
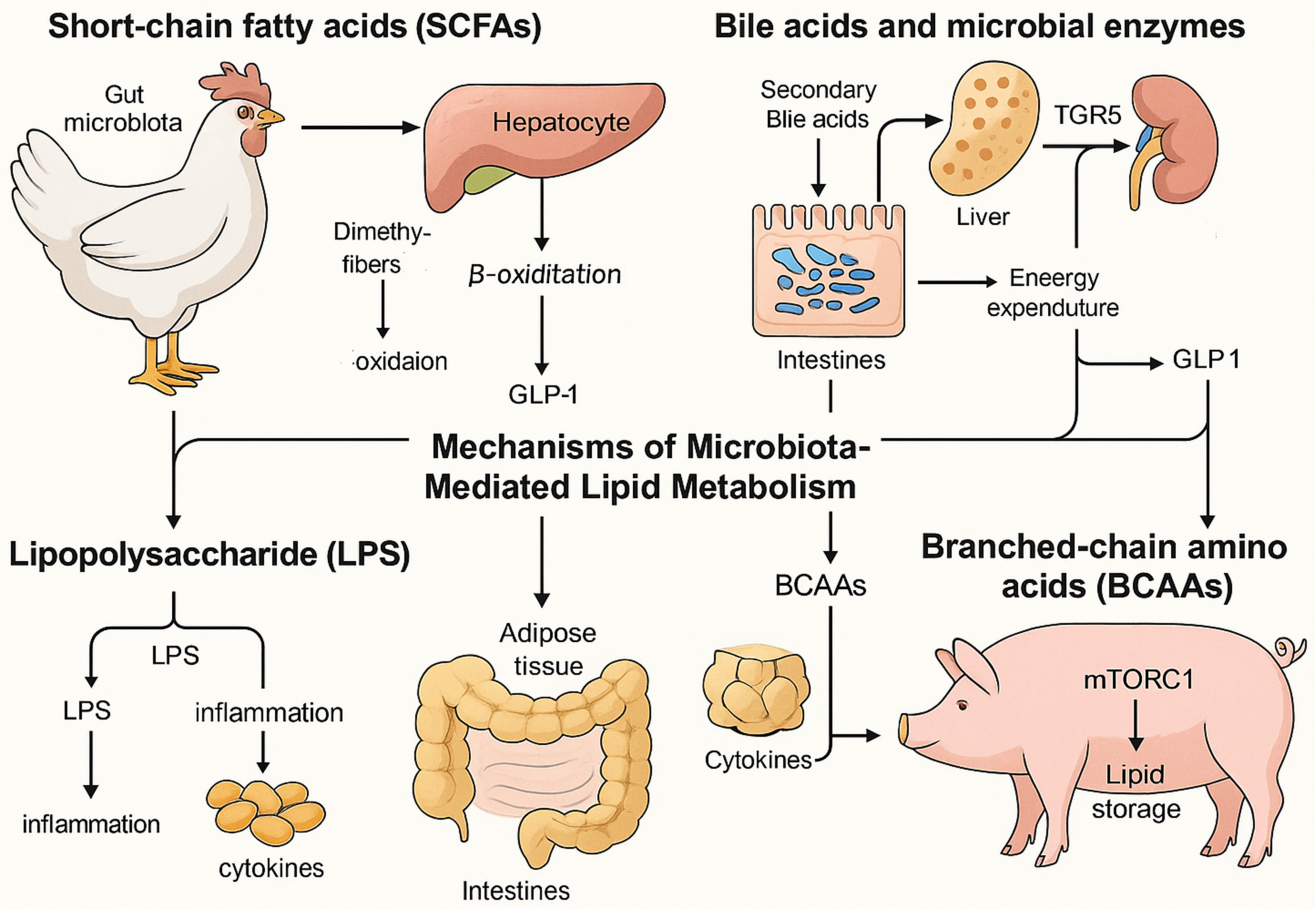
Mechanisms of gut microbiota regulation of fat deposition in chickens and pigs. This primarily includes four aspects: SCFAs inhibit lipogenesis; secondary bile acids bind TGR5 to promote thermogenesis; LPS triggers TLR4-NF-κB-driven inflammation; and BCAAs activate mTORC1 to stimulate adipogenesis. SCFAs: Short-chain fatty acids; LPS: lipopolysaccharide; NF-κB: nuclear factor-kappa B; BCAAs: branched-chain amino acids; mTORC1: mechanistic target of rapamycin complex 1.

SCFAs are produced by microbial fermentation of dietary fibers and serve as pivotal regulators of host lipid metabolism. SCFAs produced by the gut microbiota are absorbed across the intestinal epithelium and metabolized into acetyl-CoA via β-oxidation, playing a pivotal role in systemic lipid metabolism, lipogenesis, gluconeogenesis, and cholesterol synthesis^[45]^. Additionally, SCFAs function as signaling molecules by binding to and activating free fatty acid receptors (FFARs/GPRs), a class of GPCRs. This activation stimulates the secretion of glucagon-like peptide-1 (GLP-1) and modulates *de novo* lipogenesis, thereby enhancing glucose and lipid metabolism in adipose tissue and the liver^[46]^. SCFAs participate in the regulation of multiple signaling pathways associated with lipid metabolism [Figure 1]. On one hand, SCFAs modulate the transcription of key hepatic enzymes involved in lipid synthesis, such as fatty acid synthase (FAS) and acetyl-CoA carboxylase (ACC), and activate the uncoupling protein 2 (UCP2)/adenosine monophosphate-activated protein kinase (AMPK)/ACC signaling pathway, thereby promoting mitochondrial fatty acid oxidation^[47]^. On the other hand, SCFAs upregulate the expression of peroxisome proliferator-activated receptor γ coactivator-1α (PGC-1α), further activating the AMPK signaling cascade, which facilitates fatty acid oxidation while concurrently suppressing lipogenesis^[48]^. SCFAs, the principal microbial metabolites derived from colonic dietary fiber fermentation, play a pivotal role in modulating host lipid metabolism. Accumulating evidence indicates that SCFAs regulate hepatic lipid homeostasis through multiple molecular mechanisms. A critical pathway involves the inhibition of hepatic *de novo* lipogenesis via the transcriptional downregulation of sterol regulatory element binding protein-1c (SREBP-1c), the master regulator of lipogenic gene expression. By suppressing SREBP-1c-mediated lipogenic signaling, SCFAs attenuate triglyceride synthesis, thereby ameliorating hepatic steatosis and potentially preventing the pathogenesis of metabolic disorders, including NAFLD^[49]^. Overall, SCFAs serve as key metabolic integrators that bridge gut microbial activity with host lipid regulation, offering promising targets for therapeutic strategies against metabolic disorders [Figure 2].

### Bile acids and microbial enzymes

There are five main forms of bile acids: conjugated bile acids; primary bile acids, exemplified by cholic acid and chenodeoxycholic acid; secondary bile acids, predominantly represented by deoxycholic acid and lithocholic acid^[50]^. Gut bacteria express bile salt hydrolases and other enzymes that deconjugate primary bile acids and convert them into secondary bile acids, substantially altering the bile acid pool and host metabolic signaling. Secondary bile acids activate the farnesoid X receptor (FXR) in hepatic tissue to suppress lipogenesis and engage the GPCR TGR5 in adipose tissue to promote energy expenditure via thyroid hormone activation^[51,52]^. Bile acids can bind to TGR5, leading to improved insulin sensitivity, enhanced glucose tolerance, reduced plasma lipid levels, and alleviation of hepatic steatosis. TGR5 functions mainly through three pathways. First, it activates cyclic adenosine monophosphate (cAMP), which induces type 2 iodothyronine deiodinase (DIO_2_); DIO_2_ converts inactive thyroid hormone T4 into active T3, thereby promoting thermogenesis in adipose tissue^[53]^. Second, TGR5 activation in intestinal L cells promotes the secretion of GLP-1, enhancing insulin secretion and improving glucose homeostasis. Third, TGR5 modulates inflammatory responses by inhibiting the nuclear factor-kappa B (NF-κB) signaling pathway, thereby reducing inflammation associated with metabolic disorders^[54]^. Additionally, it regulates glucose metabolism and energy balance by releasing GLP-1, inhibits the NF-κB signaling pathway in macrophages, reduces foam cell formation, lowers fat deposition, and suppresses the development of atherosclerosis^[55]^. Together, these pathways highlight bile acids as critical microbial-derived regulators that orchestrate lipid metabolism, inflammation, and energy homeostasis.

### LPS

Metabolic endotoxemia, characterized by low-grade elevation of plasma LPS, triggers TLR4-NF-κB signaling in adipocytes and macrophages, driving pro-inflammatory cytokine release, insulin resistance, and adipose tissue expansion^[56,57]^. HFDs increase gut permeability, facilitating LPS translocation into the portal circulation; conversely, prebiotic and probiotic interventions that restore epithelial tight junctions lower systemic LPS levels and attenuate adipose inflammation and fat gain^[57,58]^. CD14-deficient mice resist HFD-induced weight gain and insulin resistance, confirming that LPS-CD14 interactions play a crucial role in setting the tone for metabolic inflammation and the development of obesity^[57]^. LPS triggers pro-inflammatory cytokine release, immune activation, and chronic inflammation, accelerating atherosclerosis and plaque formation. It also downregulates ATP-binding cassette transporter A1 (ABCA1) in murine macrophages, impairing cholesterol efflux. A HFD increases the abundance of LPS-producing gut bacteria, stimulating tumor necrosis factor-alpha (TNF-α) and NF-κB signaling. Both LPS and TNF-α activate apoptosis signal-regulating kinase 1 (ASK1), a critical suppressor of adipose tissue browning^[59]^. These findings underscore the pivotal role of microbiota-derived LPS in linking gut barrier dysfunction to systemic inflammation, lipid dysregulation, and metabolic disease progression.

### BCAAs

BCAAs, including leucine, isoleucine, and valine, are metabolized by both host and microbial pathways, with dysregulated microbial handling of BCAAs preceding obesity and insulin resistance^[60]^. Elevated circulating BCAAs correlate with increased fat deposition, and transplantation of BCAA-enriched microbiota from obese donors into germ-free mice raises serum BCAA levels and promotes adipocyte lipid storage^[61,62]^. The cellular uptake of BCAAs into adipocytes is predominantly regulated by specific amino acid transporters, including solute carrier family 1 member 5 (SLC1A5), solute carrier family 3 member 2 (Slc3a2), and solute carrier family 7 member 5 (Slc7a5). This complex facilitates an antiport mechanism, whereby extracellular BCAAs are exchanged for intracellular glutamine and asparagine. Upon cellular internalization, BCAAs undergo sequential metabolic transformations, culminating in the generation of intermediate acyl-CoA derivatives, including isovaleryl-CoA and 2-methylbutyryl-CoA^[63]^. These intermediates serve as critical precursors for the biosynthesis of monomethyl branched-chain fatty acids (mmBCFAs). The mitochondrial export of these acyl-CoA species is mediated by carnitine acetyltransferase, followed by their cytosolic elongation catalyzed by FAS. This metabolic cascade highlights the dual role of BCAAs in adipose tissue: not only do they serve as substrates for energy production, but they also contribute to *de novo* lipogenesis through the generation of mmBCFAs^[64]^. This pathway underscores the metabolic versatility of adipose tissue in integrating nitrogen and carbon metabolism, further emphasizing its role in systemic BCAA homeostasis and lipid biosynthesis. Green *et al.* elucidated the pivotal contribution of BCAA degradation to the control of adipocyte differentiation^[65]^. Their work revealed that increased expression of BCAA-catabolizing enzymes coincides with elevated peroxisome proliferator-activated receptor gamma (PPARγ) levels during the initial phases of adipogenesis, implicating BCAA metabolism in the determination of adipogenic fate. The study further delineated that BCAAs promote adipocyte maturation by stimulating the mechanistic target of rapamycin complex 1 (mTORC1) pathway, with ribosomal protein S6 kinase 1 (S6K1) and eukaryotic translation initiation factor 4E-binding protein 1 (4E-BP1) serving as key downstream mediators that ultimately regulate PPARγ function^[66]^. Moreover, the mitochondrial deacylase SIRT4 was identified as a modulator of BCAA metabolic flux in preadipocytes, acting through PPARγ upregulation - a finding that reinforces the critical crosstalk between mitochondrial metabolic regulation and transcriptional programming in early adipogenic commitment^[67]^. Enhancing BCAA catabolism, via dietary modulation or next-generation probiotics, improves glucose homeostasis and reduces fat mass in rodent obesity models, offering a novel avenue for metabolic health interventions^[68,69]^. Altogether, these findings highlight the integral role of BCAA metabolism in adipose tissue development, energy balance, and the pathogenesis of obesity-related metabolic disorders.

## GUT MICROBIOTA IN METABOLIC DISORDERS

### Obesity

Obese individuals and HFD rodents exhibit a characteristic shift toward an elevated Firmicutes/Bacteroidetes ratio, promoting the extraction of additional calories from complex polysaccharides and increasing fat deposition^[70]^. Concurrently, depletion of mucin-degrading and barrier-protective taxa such as *Akkermansia muciniphila* correlates with higher body mass index, while supplementation with *A. muciniphila* restores tight junction integrity and ameliorates metabolic parameters in overweight humans^[71]^. Dysbiosis also underlies metabolic endotoxemia: increased intestinal permeability allows LPS translocation into the circulation, activating TLR4-NF-κB signaling in adipose macrophages and adipocytes, driving chronic low-grade inflammation and insulin resistance^[72]^. Moreover, microbial metabolites modulate host lipid handling: SCFAs bind GPR41/43 to stimulate peptide YY and GLP-1 secretion, suppressing appetite and improving insulin sensitivity, whereas secondary bile acids generated via microbial bile salt hydrolase activity activate FXR and TGR5 to inhibit lipogenesis and enhance energy expenditure^[73,74]^. Interventions with prebiotics (e.g., inulin) increase microbial diversity and SCFA production, reducing fat mass in both murine models and clinical cohorts, underscoring therapeutic potential^[75]^.

### T2D

T2D diabetes is associated with reduced gut microbial diversity, enrichment of opportunistic pathogens, and loss of butyrate-producing taxa (e.g., *Faecalibacterium prausnitzii*), leading to impaired barrier function and systemic inflammation^[76]^. Dysbiosis also diminishes GLP-1 release: under healthy conditions, SCFAs and secondary bile acids stimulate enteroendocrine L cells to secrete GLP-1, enhancing insulin secretion and glucose tolerance; in T2D, this axis is blunted, contributing to hyperglycemia^[77]^. Probiotic and prebiotic interventions (e.g., *Lactobacillus rhamnosus*, dietary fibers) restore SCFA levels, normalize GLP-1 rhythms, and improve glycemic control in preclinical and clinical studies^[78]^. Bariatric surgery further underscores microbiota’s role: patients undergoing Roux-en-Y gastric bypass exhibit specific microbial shifts that enhance incretin responses and barrier integrity, correlating with remission of T2D^[79]^. Next-generation approaches, such as FMT from healthy donors and designer consortia, are under investigation to reprogram dysbiotic communities and reverse insulin resistance^[80,81]^.

### NAFLD

NAFLD pathogenesis is tightly linked to gut-liver axis perturbations. Dysbiosis elevates gut permeability and LPS translocation, activating hepatic TLR4-mediated inflammation and impairing mitochondrial β-oxidation^[82]^. Altered microbial metabolism of choline produces toxic intermediates (e.g., *δ-valerobetaine*) that inhibit the carnitine shuttle and exacerbate triglyceride accumulation in hepatocytes^[83]^. Conversely, SCFA-enhancing prebiotics expand myeloid-derived suppressor cells that mitigate hepatic steatosis and oxidative stress in rodent NAFLD models^[84]^. Immune crosstalk also plays a crucial role: gut-derived type 3 innate lymphoid cells migrate to the liver and secrete IL-22, promoting hepatocyte lipid oxidation and reducing fibrosis; strategies that boost the ILC3-IL-22 axis via washed microbiota transplantation have achieved up to 43% reduction in liver fat in early trials^[85,86]^. Collectively, these insights provide a mechanistic framework for microbiota-based therapies in NAFLD, including probiotics, synbiotics, and targeted microbial metabolite analogs.

### Other disorders

In addition to obesity, T2D, and NAFLD, several other well-recognized microbiota-mediated metabolic disorders have been identified, including metabolic syndrome, hypertension, and polycystic ovary syndrome (PCOS). Metabolic syndrome is associated with gut dysbiosis, reduced microbial diversity, impaired metabolism of SCFAs and bile acids, and increased levels of LPS. These alterations contribute to systemic inflammation, insulin resistance, dyslipidemia, and elevated blood pressure^[87]^. Clinical interventions using prebiotics, probiotics, synbiotics, and postbiotics have shown improvements in metabolic parameters. However, the outcomes remain variable and highlight the importance of personalized therapeutic strategies^[88]^. In hypertension, gut microbiota dysbiosis is marked by reduced microbial diversity, enrichment of mucin-degrading taxa (*Muribaculaceae*, *Alistipes*), and depletion of SCFA-producing genera (*Ruminococcus*, *Eubacterium eligens*), as observed in hypertensive cohorts. These changes correlate with altered microbial metabolic pathways (e.g., increased acetate-CoA ligase activity, decreased GPR43 signaling) and contribute to elevated blood pressure via impaired vascular and inflammatory regulation. FMT from hypertensive humans into germ-free mice has causally linked dysbiosis to hypertension development^[89,90]^. PCOS also displays characteristic features of gut dysbiosis, including lower microbial diversity, a disturbed Firmicutes to Bacteroidetes ratio, increased abundance of *Escherichia-Shigella*, and reduced levels of *Akkermansia*. These changes are correlated with insulin resistance, hormonal imbalance, and chronic inflammation^[11,91]^. Microbiota-targeted interventions, including probiotics, prebiotics, and precision microbiome-based therapies such as designer microbial consortia, have shown potential to improve both metabolic and reproductive outcomes in individuals with PCOS^[92,93]^. Table 2 summarizes these therapeutic strategies, highlighting the range of microbiota-targeted approaches being investigated for metabolic disorders.

**Table 2:** Microbiota-targeted interventions for metabolic disorders

**Disorder**	**Microbiota features**	**Mechanisms**	**Interventions**	**Reported effects and key references**
Obesity	↑ Firmicutes/Bacteroidetes, ↓ *Akkermansia*	↑ Caloric extraction, LPS-induced inflammation, ↓ barrier integrity	*Akkermansia* supplementation, prebiotics	↓ Fat mass, ↑ GLP-1, ↓ inflammation^[70-75]^
T2D	↓ Diversity, ↓ butyrate producers (e.g., *F. prausnitzii*)	↓ GLP-1 secretion, ↑ inflammation, ↓ insulin sensitivity	Pro-/prebiotics, bariatric surgery, FMT	↑ Glycemic control, ↑ GLP-1, ↓ insulin resistance^[76-81]^
NAFLD	↑ Gut permeability, ↑ LPS, altered bile acid metabolism	↑ Hepatic inflammation, ↓ β-oxidation, ↑ steatosis	Prebiotics, synbiotics, IL-22-based strategies	↓ Hepatic fat, ↓ oxidative stress, ↑ lipid oxidation^[82-86]^
Metabolic syndrome	↓ Diversity, ↑ Firmicutes/Bacteroidetes, ↑ LPS	↑ Inflammation, insulin resistance	Pre-/pro-/synbiotics	Mixed results; personalized strategies suggested^[87,88]^
Hypertension	↓ Diversity; ↑ *Muribaculaceae*, *Alistipes*; ↓ *Ruminococcus*, *Eubacterium eligens*	Impaired SCFA signaling (↓ GPR43); ↑ acetate-CoA ligase; promotes inflammation and vascular dysfunction	Probiotics, prebiotics, FMT (preclinical)	FMT from hypertensive donors induces hypertension in mice^[89,90]^
PCOS	↓ Diversity, ↑ *Escherichia-Shigella*, ↓ *Akkermansia*	↑ Insulin resistance, androgen biosynthesis, inflammation	Pro-/prebiotics, FMT, designer consortia	Improved insulin sensitivity and menstrual regulation^[91-93]^

LPS: Lipopolysaccharide; GLP-1: glucagon-like peptide-1; T2D: type 2 diabetes; FMT: fecal microbiota transplantation; NAFLD: non-alcoholic fatty liver disease; SCFA: short-chain fatty acid; PCOS: polycystic ovary syndrome.

## CONCLUSION

The gut microbiota plays a central role in regulating lipid metabolism through its metabolites and interactions with host signaling pathways. SCFAs, produced by microbial fermentation of dietary fiber, suppress hepatic lipogenesis by inhibiting SREBP-1c and activate GPR41/43 receptors to enhance mitochondrial β-oxidation. Microbial enzymes convert primary bile acids into secondary bile acids, which activate FXR to inhibit hepatic triglyceride synthesis and stimulate TGR5 receptors to promote adipose thermogenesis. LPS translocated from dysbiotic microbiota triggers TLR4-NF-κB signaling, driving insulin resistance and adipose tissue inflammation. BCAAs, metabolized by gut microbes, activate the mTORC1-PPARγ axis to promote adipogenesis, with elevated circulating BCAA levels strongly linked to obesity and metabolic dysfunction. In livestock, microbiota-targeted strategies optimize fat deposition for meat quality. For instance, probiotic supplementation in pigs enriches *Lactobacillus* and BCAA metabolic pathways, enhancing IMF, while dietary succinate or coated sodium propionate reshapes cecal microbiota in chickens to reduce abdominal fat. FMT experiments confirm the causal role of microbial communities in transferring fat deposition phenotypes. Furthermore, the conserved mechanisms linking dysbiosis to metabolic disorders (obesity, T2D, NAFLD) across species highlight the dual role of microbial metabolites: SCFAs and secondary bile acids ameliorate metabolic inflammation, whereas LPS and BCAA imbalances exacerbate lipid dysregulation.

Future research should prioritize precision interventions that target specific microbial pathways - such as engineered probiotics, metabolite analogs, and bacteriophage therapy - to enhance both livestock productivity and human metabolic health. The integration of multi-omics approaches (e.g., metagenomics, metabolomics, transcriptomics) will be essential to uncover functional microbial targets and accelerate the development of effective microbiota-based therapies. However, translating these strategies into clinical and agricultural applications remains challenging due to individual variability in microbiota responses and concerns regarding the long-term safety and stability of engineered microbial consortia.

## DECLARATIONS

### Copyright

© The Author(s) 2025.
